# Chronic Cough and Causes in Children

**DOI:** 10.3390/jcm12123947

**Published:** 2023-06-09

**Authors:** Miles Weinberger

**Affiliations:** 1 Respiratory Department, Rady Children’s Hospital, University of California San Diego, La Jolla, CA 92093, USA; miles-weinberger@uiowa.edu; 2 Pediatric Department, University of Iowa, Iowa City, IA 52242, USA

## 1. Cough

Coughing is a natural means to clear the airway. When a cough is chronic and present for more than 4 weeks in a child, it can be from aspiration of a foreign substance, a respiratory infection, an anatomical abnormality, or a defect in innate or acquired immunity. Coughing can also occur when there is a perception, a feeling, of something in the airway when no object is there. What are the causes a physician may encounter in a child with a chronic cough?

## 2. Causes

**Viral respiratory infections** are a common cause of acute coughs. Particularly frequent in pre-school age children, coughs from viral respiratory infections are generally self-limited. However, some cause sufficient concern for parents that they will seek medical care. It was reported that 20% of young children, with a mean age 2.3 years, seen at an emergency department (ED) in Brisbane Australia were still coughing 4 weeks after being seen in the ED. About 2/3 of these children were evaluated by a pediatric pulmonologist who found protracted bacterial bronchitis in almost half of the patients and a new diagnosis in others, including asthma, bronchiectasis, and tracheobronchomalacia [1].

**Protracted bacterial bronchitis** (PBB) can result from an acute cough. PBB is the most common cause of chronic cough in infants and toddlers [2]. Although generally responsive to 14 days of amoxicillin clavulanate, half of the children identified with PBB have recurrences [3] and 8% have been reported to progress to bronchiectasis [4].

**Asthma** is another common cause of a chronic cough that can occur at all ages. Although asthma is classically associated with wheezing, coughing as a symptom is almost as frequent as wheezing. Sometimes, a cough is the only apparent symptom, and the patient is diagnosed with cough variant asthma. However, other components of asthma are usually present, and responsiveness to bronchodilators or corticosteroids readily distinguishes this cause of chronic cough. Although asthma is likely to be diagnosed by a primary care physician, referral for a chronic cough was described in a multicenter study as second in frequency only to PBB.

**Figure 1:**
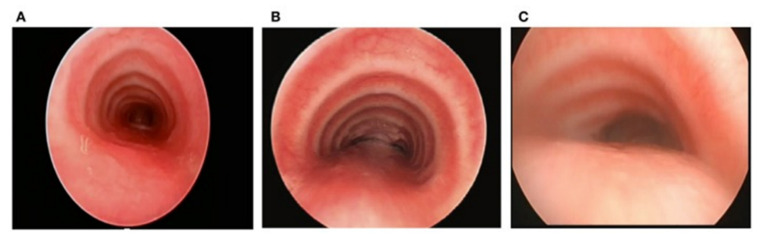
Normal C-shaped cartilaginous rings in (**A**) result in the trachea maintaining its lumen even when coughing causes protrusion of the membrane. (**B**) illustrates U-shaped cartilaginous rings, which result in a broader membranous portion that can protrude more with increased thoracic pressure during a cough. (**C**) shows bow-shaped cartilaginous rings that would result in a high likelihood that the membranous portion would come in contact with the cartilaginous rings, causing a cough and interfering with the normal mucociliary clearance of mucus.

**Figure 2:**
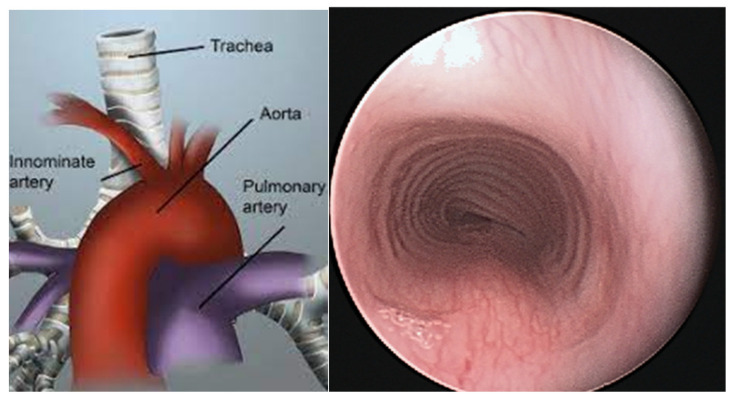
The figure on the (**left**) shows the innominate artery crossing over the trachea where it might compress that portion of the trachea. The figure on the (**right**) shows compression from the upper right that sometimes results in less rigidity of the cartilaginous rings at that site.

**Tracheobronchomalacia** is sometimes confused with asthma because of wheezing from a narrowed airway. The European Respiratory Society has defined tracheal or bronchial malacia as a greater than 50% reduction in the cross-sectional area during expiration [5]. Moderate and severe tracheomalacia were defined as 75% and 100%, respectively. Airway malacia can be associated with other congenital anomalies. The presence of tracheomalacia or bronchomalacia is commonly not associated with any symptoms. However, in addition to the presence of a barking cough, tracheomalacia can cause noisy breathing, wheezing, dyspnea, and even dying spells in infants. Collapse of the lumen of the trachea or bronchus can be from a large pliable membranous portion of the trachea (Figure 1) or from compression from an artery. An example is where the innominate artery crosses the trachea (Figure 2).

The incidence of tracheomalacia in the general population of the Netherlands is estimated to be at least 1 in 2100 births [6]. Mild to moderate airway malacia found in infants and toddlers is reported to frequently be outgrown by 2 years of age. A hospital in the Netherlands examined 512 bronchoscopies that had been performed over seven years. Primary airway malacia without a concurrent medical condition was observed in 136 patients, 46% with tracheomalacia, 36% with both tracheomalacia and bronchomalacia, and 18% with just bronchomalacia. Bronchomalacia, when present, occurred bilaterally in 26% of patients, on the right only in 45% and on the left only in 29%. Tracheal and bronchomalacia are also associated with protracted bacterial bronchitis, which is an important cause of coughing in infants and toddlers. 

Tracheomalacia can be associated with a characteristic cough that might be confused with a habit cough. Robert Wood described seven children, ages 7 to 13, with severe non-productive intractable coughs of four to twenty-four months duration. An illness consistent with a viral respiratory infection was an initiating factor in all the patients. Coughing was described as paroxysmal throughout waking hours and during sleep [7]. Because their previous evaluation identified no explanation, flexible bronchoscopy was performed with topical anesthesia and light sedation. Tracheomalacia was apparent in six patients and right mainstem bronchomalacia was present in one patient. During quiet breathing, the airways looked normal, but coughing was associated with complete localized total collapse of the lumen with the membranous portion touching the central cartilaginous portion and vibrating. Five of the seven had cessation of coughing with hypnosis, suggesting they may also have had a component of habit cough. The other two patients did not return for follow-up and were still coughing when contacted.

**Figure 3:**
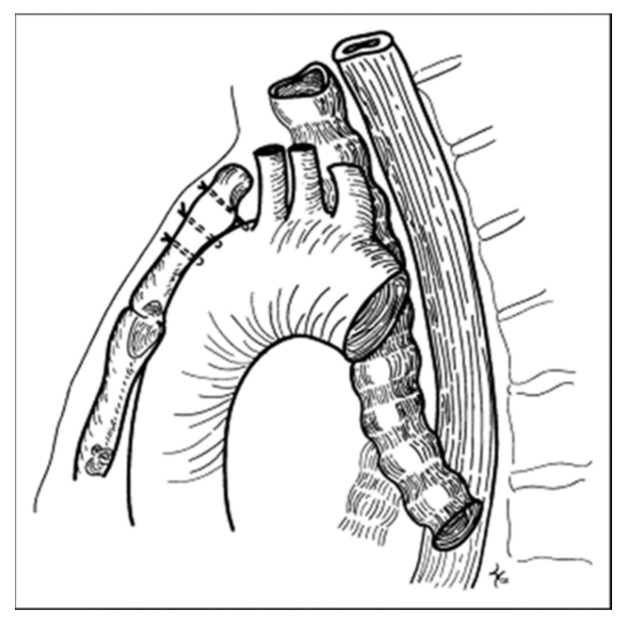
Anterior aortopexy where stitches through the intima of the aorta and the sternum pull the main vessels and the innominate artery away from the trachea, thereby eliminating the area of compression.

A more severe result of tracheomalacia-induced cough was observed in a 9-year-old girl with 2 years of a harsh, dry, barking cough. It was paroxysmal with frequent post-tussive emesis. She experienced nightly awakening from the cough and had been hospitalized several times because of the intractable coughing. Treated for presumed asthma, she received sufficient prednisone to cause growth suppression and Cushingoid changes in appearance. The continued cough despite the prednisone justified a flexible bronchoscopy with topical anesthesia and light sedation. A broad membranous portion of the trachea and right upper compression at the mid-trachea resulted in tracheal collapse during expiration at that site. An anterior aortopexy was performed (Figure 3) with relief of the coughing and cessation of the prednisone.

**Primary ciliary dyskinesia** is a congenital disorder caused by genetic mutations that affect the mucosal cilia structure of the airways and elsewhere [8]. Transient neonatal respiratory distress is common, and a cough is a major symptom with onset during infancy. Because the disorder affects several proteins of the cilia, this dysfunction can result in immobility of the cilia or cilia movement that is not coordinated to provide normal mucociliary clearance. The result is a chronic wet cough. Also involved is chronic rhinorrhea and recurrent otitis media. Situs inversus is present in about 50% of those with primary ciliary dyskinesia and the combination is known as Kartagener’s syndrome.

Mutations of 50 genes causing primary ciliary dyskinesia have been reported [9]. However, genetic diagnosis continues to be limited, as disease-causing mutations are not always identified in those with the abnormality [10]. Diagnosis often requires multiple tests including nasal nitric oxide, electron microscopy, high-speed video microscopy analysis, and immunofluorescence, in addition to genotyping. The level of nasal nitric oxide in these patients is about 10 times lower than healthy controls. Electron microscopy examines the structure of the cilia and identifies various abnormalities of the structure of the nine doublet outer and a pair of two central microtubules, but normal structures are observed in some patients. Immunofluorescence directed against ciliary proteins has been useful for diagnosis. The presence of a chronic wet cough and development of non-cystic fibrosis bronchiectasis warrants consideration of primary ciliary dyskinesia.

Treatment focuses on the pulmonary symptoms that result in recurrent or chronic infections with eventual bronchiectasis. The most important treatment is probably chest physical therapy for airway clearance to compensate for the lack of normal mucociliary clearance and judicious use of antibiotics. However, there are no controlled clinical studies that document any specific treatment for children. The lung clearance index measured by multiple-breath washout represents a sensitive method for detecting and monitoring disease in those with primary ciliary dyskinesia. Survival is variable with the possibility of a near-normal life span occurring in some patients.

**Aspiration** of foreign bodies can be retained and then can result in a persistent cough, often with wheezing. A dramatic case has been reported of an 8-year-old girl with a chronic cough that persisted for 15 months before a wheat sheaf was identified in a lower lobe bronchus of the right lung [11]. In a report on the late diagnosis of foreign body aspiration in children, a cough was the most frequent symptom, present in 29 of 32 children [12]. Diagnosis is generally achieved using the patient’s history, confirmed radiologically or by direct observation during bronchoscopy. Treatment includes the removal of the foreign body and treatment of any persistent infection.

**Figure 4:**
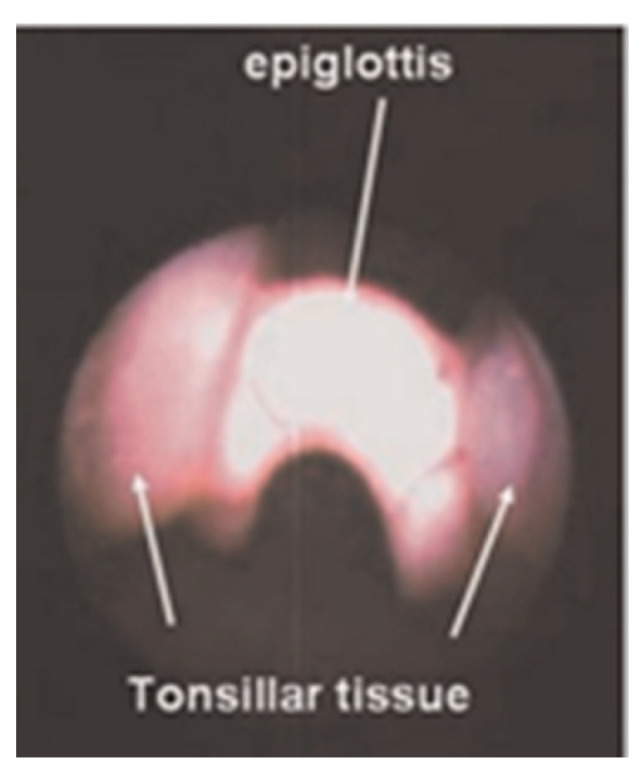
Tonsils impinging on the epiglottis in a 5-year-old girl with a chronic cough.

**Figure 5:**
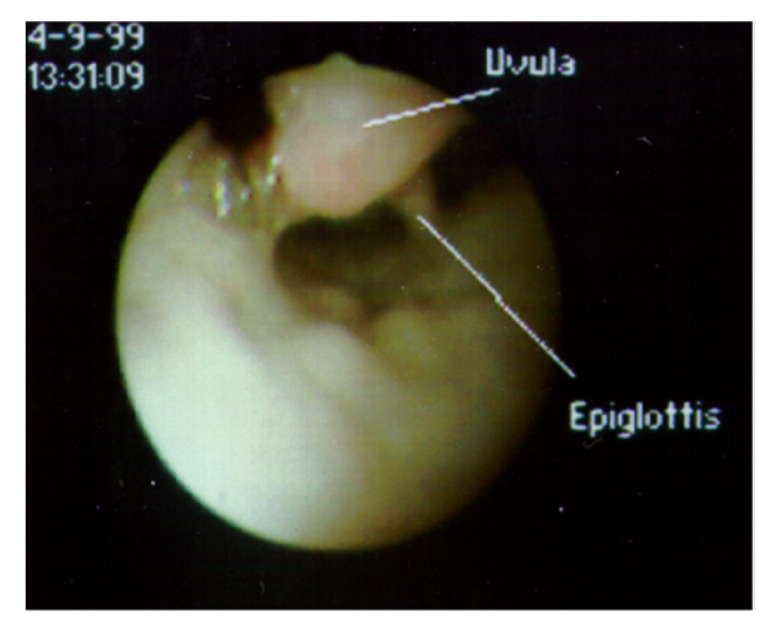
View of supraglottic area through a flexible fiberoptic bronchoscope inserted through the nose, with the patient supine. The uvula, which cannot normally be observed when the epiglottis is visualized, is lying in contact with the epiglottis.

**Abnormalities of pharyngeal structures** can cause a chronic cough. Tonsils have been associated with a persistent cough when they impinge upon the epiglottis (Figure 4) [13]. The uvula also has been reported to cause a cough when of sufficient length or positioned in such a way that it comes in contact with the epiglottis (Figure 5) [14]. A nocturnal cough is common with both of these causes of cough. When a chronic cough is not associated with any apparent lung disease and does not fit the pattern of a habit cough, flexible bronchoscopy through the nose permits visualization of the upper airway prior to progression to the lower airway. While the tonsils or uvula are not ordinarily a cause of coughing, visualizing their atypical contact with the epiglottis is sufficient to suspect that those observations are the cause of chronic coughing in these children. Treatment includes tonsillectomy or uvulectomy to eliminate the stimulation on the epiglottis. Postoperative cessation of coughing supports this diagnosis.

**Arnold’s nerve reflex** is a cough induced by stimulating the ear canal. There have been a hundred years of occasional case reports of chronic coughing from a foreign body in the ear. One adult case report of a chronic cough stopped upon removing a hair in the ear canal [15]. The frequency that an Arnold nerve cough reflex can be demonstrated in normal adults and children is about 2%. Inducing an Arnold’s nerve reflex in 100 adults and children with a chronic cough by stimulating the external ear canal was reported to be effective in 23% of adults with a chronic cough and 3% of children with a chronic cough [16]. While chronic coughing from a persistent foreign body in the ear is rare, otoscopic ear examination is justified in the evaluation of patients with a chronic cough.

**A habit cough** is a cough without a cause. It is a repetitive daily dry cough that is absent during sleep and has previously been described in detail [17]. Its prevalence at two major referral centers was about 9 patients per year. In a multicenter study of 346 children referred for a chronic cough, 101 children did not have PPB, asthma, or spontaneous resolution; 15 of those 101 were diagnosed with a habit cough. The character of the cough can be similar to that of tracheomalacia but the total absence during sleep is a distinguishing characteristic. The diagnosis of a habit cough is not one of exclusion. Rather, the diagnosis is made by its unique clinical presentation.

**The controversial causes of persistent cough in children** include asthma, post-nasal drip, and gastroesophageal reflux (GERD). Asthma can cause a chronic cough but should not be considered if other symptoms of asthma are not present, and the cough does not respond to asthma medications. A short course of an oral corticosteroid can be a diagnostic test if necessary [18]. The medical literature on chronic coughing contains claims of postnasal drip, renamed the upper airway cough syndrome, and gastroesophageal reflux. There is no support for these in children; there are no clinical trials justifying consideration of these hypothesized etiologies and there is published skepticism [19,20,21]. While postnasal drip and gastroesophageal reflux are common in children with chronic coughing, there is no evidence supporting causation.

## 3. Summary

The causes of chronic cough addressed in this chapter include tracheomalacia, primary ciliary dyskinesia, retained aspiration, pharyngeal abnormalities Arnold’s reflex, and habit coughing. A habit cough is diagnosed by its unique clinical presentation without diagnostic tests. Patient history, radiology, and flexible bronchoscopy can usually distinguish the other causes of chronic cough discussed in this chapter.
